# Multiple Oral Manifestations in a Patient With Chronic Graft Versus Host Disease: A Case Report

**DOI:** 10.7759/cureus.70233

**Published:** 2024-09-26

**Authors:** Duo Li, Yidan Shan, Weilian Sun, Xiangjian Wang

**Affiliations:** 1 Department of Oral Medicine, The Affiliated People's Hospital of Ningbo University, Ningbo, CHN; 2 Department of Oral and Maxillofacial Surgery, The Second Affiliated Hospital of Zhejiang University School of Medicine, Hangzhou, CHN; 3 Department of Oral Medicine, The Second Affiliated Hospital of Zhejiang University School of Medicine, Hangzhou, CHN

**Keywords:** early intervention, leukoplakia, oral cancer, oral chronic graft-versus-host disease, salivary dysfunction

## Abstract

Hematopoietic stem cell transplantation (HSCT) is the standard treatment for hematopoietic malignancies and certain solid tumors; however, graft-versus-host disease (GVHD) remains a significant complication. Chronic GVHD (cGVHD), which occurs more than 100 days post-transplant, can lead to various oral manifestations that necessitate multidisciplinary management to prevent disease progression and enhance the quality of life. We present the case of a 60-year-old woman who developed dry mouth, oral pain, a brown-yellow tongue, and dental caries seven years after receiving HLA-matched unrelated donor HSCT. Examination revealed a brown-yellow hairy tongue, extensive tooth decay, reduced saliva production, and oral leukoplakia with mild epithelial hyperplasia, along with dysfunction of the salivary and lacrimal glands. Following appropriate treatment, the patient’s symptoms resolved without recurrence at the six-month follow-up. This case highlights the importance of professional diagnosis, timely interventions, and regular monitoring in managing oral cGVHD to achieve a favorable prognosis.

## Introduction

Chronic graft-versus-host disease (cGVHD) is a major cause of morbidity and mortality following allogeneic hematopoietic stem cell transplantation (HSCT) [1]. It is an autoimmune condition driven by an immune response, where donor T cells recognize and attack antigens expressed by normal tissues post-HSCT due to minor histocompatibility antigen disparities between donors and recipients [2,3].

More than 80% of patients with cGVHD experience oral involvement, commonly presenting as lichenoid mucositis, immune-mediated salivary gland dysfunction, and tissue fibrosis. These complications can cause sensitivity to spicy foods, dry mouth, speech difficulties, swallowing and chewing problems, and halitosis. These symptoms often lead to reduced oral intake, malnutrition, an increased risk of oral infections, higher utilization of medical services, and a significant decline in patients’ health-related quality of life [4,5]. Notably, the presence of cGVHD in the oral cavity may increase the risk of oral cancer, highlighting the need for early intervention in oral disease management [6,7].

We present a rare case of oral cGVHD in a patient who had acute myeloid leukemia seven years prior and underwent allogeneic HSCT from a matched sibling donor. The cGVHD in this case is characterized by a hairy tongue, widespread dental caries, salivary and lacrimal gland dysfunction, and leukoplakia lesions.

This article was previously posted to the medRxiv preprint server on June 7, 2024.

## Case presentation

A 60-year-old woman presented to the Department of Oral Medicine in January 2023 with oral pain that had persisted for the past two weeks, significantly affecting her ability to eat, speak, and sleep. She also reported a six-month history of dry mouth (xerostomia) and dry eyes, without experiencing fatigue or musculoskeletal pain. The patient denied using tobacco or alcohol and reported no recent exposure to oral rinses or antibiotics. She had no history of head and neck radiotherapy and was not taking medications known to affect salivary gland function, such as atropine, acetaminophen, or scopolamine. However, she had undergone HLA-matched allogeneic HSCT for acute myeloid leukemia seven years earlier. To prevent GVHD, she was administered intravenous tacrolimus at a dose of 0.03 mg/kg 24 hours before transplantation, along with short-term methotrexate. After transplantation, she received oral tacrolimus at a 1:4 ratio under medical supervision once daily for a total of five years.

**Figure 1:**
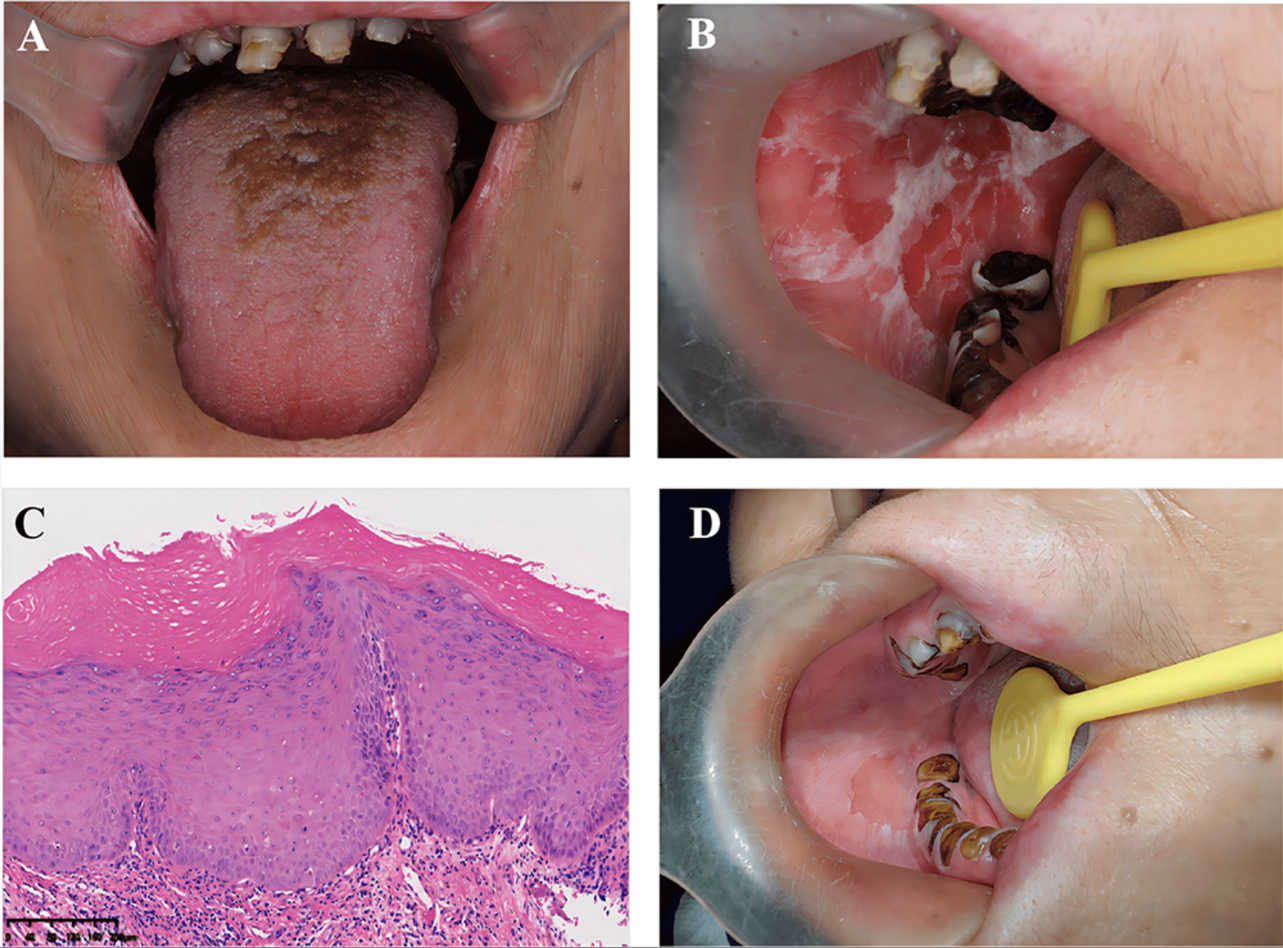
Oral presentations and pathological examination results of the patient. A: A brown-yellow hairy tongue and extensive caries were visible. B: Widespread white plaque, erythematous, and erosive lesions on the right buccal mucosa were seen. C: Hematoxylin and eosin staining revealed the pathological characteristics of leukoplakia with mild dysplasia. D: After six months, clinical images showed that the erosion had healed, and the white plaque lesion had thinned.

**Figure 2:**
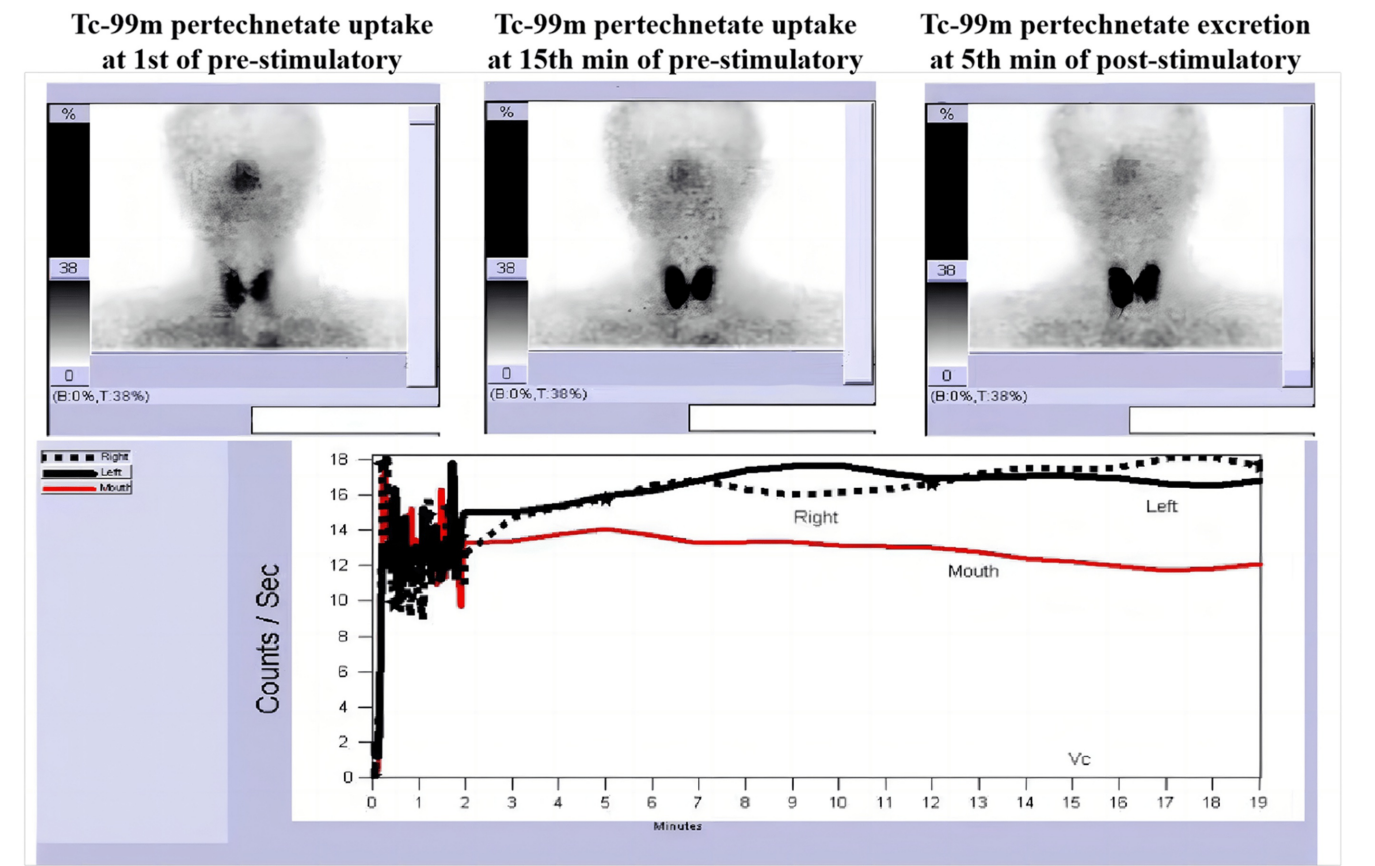
Salivary gland scintigraphy examination. There was hardly any uptake/excretion of Tc-99m pertechnetate from pre-stimulatory 15 minutes to post-stimulatory five minutes.

Clinical examination revealed a brown-yellow hairy tongue, extensive dental caries (Figure 1A), reduced saliva production upon salivary gland compression, and heterogeneous lesions on the right cheek mucosa, including white patches with areas of atrophy and ulceration (Figure 1B). Salivary gland scintigraphy with Tc-99m showed decreased uptake and excretion (Figure 2), indicating severe salivary gland dysfunction. Antinuclear antibody testing was positive with a titer of 1:40. Schirmer’s test confirmed lacrimal gland dysfunction, with a wet filter paper length of 1 mm/five minutes for both eyes. Histopathological examination of the right cheek mucosa revealed oral leukoplakia with a risk of malignant transformation (Figure 1C). 

Based on these findings, the patient was diagnosed with cGVHD presenting as oral leukoplakia and immune-mediated salivary dysfunction. Given the mild hyperplasia grade of the oral leukoplakia, surgical excision was not recommended. Treatment options included artificial saliva and tear solutions for gland dysfunction, dexamethasone 0.01% mouthwash and sodium bicarbonate 2% solution for oral mucosal inflammation, and oral β-carotene soft capsules (6 mg daily) for the prevention of malignant transformation in oral leukoplakia. Regarding the patient's dental caries issue, we referred her to the Department of Conservative Dentistry and Endodontics for caries restoration or root canal treatment, followed by routine cleaning and fluoride application.

After one month, the patient showed significant improvement in dryness and pain symptoms. This improvement persisted for three months without any adverse events or unexpected incidents during the treatment. At the most recent six-month follow-up, the patient did not report any significant clinical discomfort, and there were no issues affecting her daily life (Figure 1D). The patient provided written informed consent for the publication of this case report and accompanying images.

## Discussion

A 63-year-old female presented with symptoms of oral cGVHD seven years after allogeneic HSCT, including a hairy tongue, extensive dental caries, decreased saliva, and white patchy lesions. Patients with oral cGVHD experience higher rates of late-onset complications and mortality, and they have lower overall survival and quality of life compared to transplant recipients without oral cGVHD [8].

The diagnosis of oral cGVHD is based on the assessment of clinical symptoms, including oral pain, ulcers, patches, dryness, decreased appetite, and changes in the oral mucosa. Taking a detailed history and carefully observing the patient’s oral symptoms are important initial steps in the diagnosis. Biopsy tissue examination helps identify pathological features, while blood and saliva tests provide supportive evidence [9].

Salivary dysfunction is a common feature of cGVHD, driven by adaptive immune responses targeting host tissues, leading to tissue fibrosis similar to autoimmune diseases like Sjögren’s syndrome. Symptoms such as xerostomia, dry eye syndrome, and auxiliary test results suggest that our patient exhibits characteristics similar to Sjögren's syndrome, indicating potential autoimmune involvement in salivary gland dysfunction due to cGVHD [10]. Salivary dysfunction exacerbates oral health issues, including dental caries and hairy tongue. Prompt improvement of oral lubrication is essential to alleviate dry mouth and reduce the risk of complications. Although our patient did not exhibit oral mucosal lesions resembling lichen planus, lichenoid changes are common in oral cGVHD and are characterized by white stripes or patches with erythema and ulceration [11].

Scholars suggest that cGVHD may manifest in the oral mucosa as white lesions, erythema, and ulcers. The minimum histological criteria for diagnosing oral cGVHD include the presence of lymphocytic infiltration within the mucosa [12]. Moreover, studies indicate that patients with oral epithelial atypical hyperplasia following allogeneic HSCT are at an increased risk of developing oral malignancies [13]. Therefore, early biopsy of oral white patches in patients who have undergone allogeneic HSCT is recommended to assess the risk of malignant transformation, which is crucial for improving patient prognosis [14]. In this case, the patient’s pathology revealed oral leukoplakia with mild epithelial atypical hyperplasia, suggesting a relatively low risk of cancer. In addition, the presence of extensive lymphocytic infiltration in the lamina propria supports the diagnosis.

Treatment for oral cGVHD varies, with local intervention being the main approach, especially for patients experiencing pain and reduced oral function. Furthermore, the oral cavity is one of the few organs where intensive topical therapy can effectively treat moderate to severe conditions [4]. The use of calcineurin inhibitors and immunosuppressive therapies carries the risk of secondary fungal infections and malignant transformation [15,16]. Hence, alongside surgical excision, we provided local dexamethasone mouthwash and sodium bicarbonate solution without involving other systemic immunosuppressive drugs in our case, which showed a relatively significant treatment effect. In addition, we administered oral beta-carotene to mitigate the risk of malignant transformation. Progressive dental caries in cGVHD patients increase the risk of infections and dental care costs. It is recommended to limit sugar intake, use fluoride gel, and maintain regular follow-ups for oral hygiene [10]. The patient in our case was satisfied with the treatment outcomes and was willing to cooperate with oral hygiene maintenance.

## Conclusions

The evidence suggests that the oral manifestations of cGVHD are interconnected, affecting not only the quality of life for patients but also posing a risk of malignant transformation for certain complications. This underscores the importance of regular professional examination and local management of these lesions. It is advisable to provide a comprehensive team, including oral medicine experts, to longitudinally monitor cGVHD patients with oral involvement. This approach aims to track disease progression and ensure the overall health of the patients.
